# NT‐proBNP is a weak indicator of cardiac function and haemodynamic response to exercise in chronic heart failure

**DOI:** 10.1002/ehf2.12424

**Published:** 2019-02-20

**Authors:** Milos Parovic, Nduka C. Okwose, Kristian Bailey, Lazar Velicki, Zlatko Fras, Petar M. Seferovic, Guy A. MacGowan, Djordje G. Jakovljevic

**Affiliations:** ^1^ Cardiovascular Research Centre, Institute of Cellular Medicine Newcastle University Newcastle upon Tyne UK; ^2^ Institute of Genetic Medicine, Faculty of Medical Sciences Newcastle University Newcastle upon Tyne UK; ^3^ Newcastle upon Tyne Hospitals NHS Foundation Trust Newcastle upon Tyne UK; ^4^ Faculty of Medicine University of Novi Sad Novi Sad Serbia; ^5^ Department of Cardiovascular Surgery Institute of Cardiovascular Diseases Vojvodina Sremska Kamenica Serbia; ^6^ Department of Vascular Diseases, Division of Internal Medicine University Medical Center Ljubljana Ljubljana Slovenia; ^7^ Department of Internal Medicine, Faculty of Medicine University of Ljubljana Ljubljana Slovenia; ^8^ Cardiology Department, Medical School University of Belgrade Belgrade Serbia; ^9^ Clinical Centre Serbia Belgrade Serbia; ^10^ Serbian Academy of Science and Arts Belgrade Serbia; ^11^ RCUK Centre for Ageing and Vitality Newcastle University Newcastle upon Tyne UK

**Keywords:** Heart failure, NT‐proBNP, Cardiac power, Exercise

## Abstract

**Aims:**

N‐terminal prohormone of brain natriuretic peptide (NT‐proBNP) plays an important role in diagnosis and management of heart failure. The aim of the present study was to assess haemodynamic response to exercise and to evaluate the relationship between NT‐proBNP, cardiac function, and exercise tolerance in chronic heart failure.

**Methods and results:**

A single‐centre, cross‐sectional pilot study recruited 17 patients with chronic heart failure with reduced left ventricular ejection fraction (age 67 ± 7 years) and 20 healthy volunteers (age 65 ± 12 years). The NT‐proBNP was measured in the heart failure group. All participants completed maximal graded cardiopulmonary exercise stress testing coupled with gas exchange (using metabolic analyser for determination of exercise tolerance, i.e. peak O_2_ consumption) and continuous haemodynamic measurements (i.e. cardiac output and cardiac power output) using non‐invasive bioreactance technology. Heart failure patients demonstrated significantly lower peak exercise cardiac function and exercise tolerance than healthy controls, i.e. cardiac power output (5.0 ± 2.0 vs. 3.2 ± 1.2 W, *P* < 0.01), cardiac output (18.2 ± 6.3 vs. 13.5 ± 4.0 L/min, *P* < 0.01), heart rate (148 ± 23.7 vs. 111 ± 20.9 beats/min, *P* < 0.01), and oxygen consumption (24.3 ± 9.5 vs. 16.8 ± 3.8 mL/kg/min, *P* < 0.01). There was no significant relationship between NT‐proBNP and cardiac function at rest, i.e. cardiac power output (*r* = −0.28, *P* = 0.28), cardiac output (*r* = −0.18, *P* = 0.50), and oxygen consumption (*r* = −0.18, *P* = 0.50), or peak exercise, i.e. cardiac power output (*r* = 0.18, *P* = 0.49), cardiac output (*r* = 0.13, *P* = 0.63), and oxygen consumption (*r* = −0.05, *P* = 0.84).

**Conclusions:**

Lack of a significant and strong relationship between the NT‐proBNP and measures of cardiac function and exercise tolerance may suggest that natriuretic peptides should be considered with caution in interpretation of the severity of cardiac dysfunction and functional capacity in chronic heart failure.

## Introduction

Heart failure is a clinical syndrome associated with poor prognosis, characterized by reduced cardiac output at rest and/or stress.^1^ Natriuretic peptides are widely used in clinical practice to help the diagnosis of heart failure, but the low specificity limits their diagnostic utility.^2^ Daily clinical practice confirms limited accuracy of increased serum levels of natriuretic peptides to predict the presence of heart failure. Despite these limitations, natriuretic peptides continue to be used in heart failure diagnostic pathway.^1^ The literature report on the relationship between natriuretic peptides and cardiac function and performance in response to stress is limited. Better understanding of this relationship is important as it would allow for more accurate monitoring and risk stratification in heart failure. The aim of the present study was to assess haemodynamic response to exercise and to evaluate the relationship between N‐terminal prohormone of brain natriuretic peptide (NT‐proBNP), cardiac function, and exercise tolerance in chronic heart failure.

## Methods

### Study design

This was a single‐centre, cross‐sectional pilot study to assess the relationship between NT‐proBNP and cardiac function and exercise tolerance.

### Participants

The study included 17 patients with chronic heart failure and 20 healthy individuals (*Table* 1). The study inclusion criteria for heart failure were adults (i) >50 years of age, (ii) diagnosed with stage II–III New York Heart Association functional class of chronic heart failure due to left ventricular systolic dysfunction (<40%), (iii) had NT‐proBNP >125 ng/L, (iv) were clinically stable for at least 6 weeks prior to screening, and (v) were optimally treated for chronic heart failure. The exclusion criteria were (i) inability to provide informed consent, (ii) inability to perform the exercise stress testing, (iii) serious co‐morbidities, i.e. severe aortic stenosis or uncontrolled arrhythmias, (iv) myocardial intervention/coronary artery bypass grafting in the past 3 months, (v) severe obesity (body mass index > 40 kg/m^2^), and (vi) patients in New York Heart Association class I or IV. The ‘healthy control’ group consisted of age‐matched healthy individuals.

**Table 1:** Participants' demographics and physical characteristics

	Healthy controls (*n* = 20)	HF patients (*n* = 17)	*P*‐value
Age (years)	65 ± 12	67 ± 7	0.57
Men/women (*n*)	13/7	13/4	—
Weight (kg)	76 ± 13	84 ± 15	0.08
Height (cm)	165 ± 8.8	172 ± 5.8	0.01
Body surface area (m^2^)	1.83 ± 0.2	1.97 ± 0.2	0.02
Body mass index (kg/m^2^)	27.7 ± 3.3	28.2 ± 4.5	0.73
Aetiology Ischaemic heart disease/dilated cardiomyopathy	—	13/4	—
Left ventricular ejection fraction (%)	—	32 ± 7.5	—
NT‐proBNP level (ng/L)	—	420 ± 265	—
Medication	—		—
Beta‐blockers	—	17	—
Angiotensin‐converting enzyme inhibitors	—	15	—
Angiotensin receptor blockers	—	2	—
Diuretics	—	13	—
Warfarin	—	6	—
Anti‐arrhythmic	—	3	—

HF, heart failure; NT‐proBNP, N‐terminal prohormone of brain natriuretic peptide.

### Procedures

The patients in the ‘heart failure’ group underwent blood sampling for determination of plasma NT‐proBNP. Subsequently, all participants completed maximal graded cardiopulmonary exercise stress testing using a semi‐recumbent, electromagnetically controlled cycle ergometer (Corrival; Lode, Groningen, the Netherlands). Bioreactance method (NICOM, Cheetah Medical, USA) and gas exchange monitoring (Metalyzer 3B, Cortex, Leipzig, Germany) were used to non‐invasively assess cardiac output and gas exchange. Bioreactance has been validated against gold‐standard measures of cardiac function and has been shown to be a reliable and valid method of estimating cardiac output.^3^, ^4^, ^5^

Incremental exercise stress testing protocol was used, where patients were required to maintain cycling speed between 60 and 70 rpm, with work increasing by 10 W/min. Standardized Borg scale was used to assess the perceived exertion during exercise (6—no exertion at all and 20—maximal exertion).^6^ The test was terminated when (i) the respiratory exchange ratio showed the maximal level of exertion (respiratory exchange ratio > 1.15), (ii) a patient was unable to maintain required cycling cadence, or (iii) until patients reached volitional exertion and desired to stop. Cardiac power output, as an integrative and direct measure of overall function and pumping capability of the heart, was calculated as a product of simultaneously measured mean arterial blood pressure and cardiac output.^7^, ^8^

### Statistical analysis

Data were analysed using SPSS version 24.0 (SPSS Inc., Chicago, IL, USA). All data were screened for univariate outliers using *Z*‐distribution cut‐off scores and Mahalanobis distance test for multivariate outliers. Independent samples *T*‐tests were used to assess the differences in physiological variables between patients with heart failure and healthy controls. The relationship between NT‐proBNP and measures of cardiac function and exercise tolerance was evaluated using Pearson's product moment correlation coefficient analysis (*r*). Coefficient of determination (*R* ^2^) was used to determine the goodness of fit within the correlation. Statistical significance level was set to *P* ≤ 0.05. All data are presented as mean ± standard deviation.

## Results

### Demographics and physical characteristics

Study participants' demographics and physical characteristics are presented in *Table* 1. Body mass index and age were not significantly different between the groups (*P* > 0.05). The mean left ventricular ejection fraction in patients with chronic heart failure was 32 ± 7%, and mean NT‐proBNP level was 420 ± 265 ng/L. Patients were treated with a recommended medical therapy including beta‐blockers, angiotensin‐converting enzyme inhibitors (or angiotensin receptor blockers), and diuretics as appropriate.

### Difference in cardiac function at rest

*Table* 2 demonstrates haemodynamic and metabolic measures for heart failure patients and healthy controls, at rest. Systolic, diastolic, and mean arterial blood pressures were significantly higher in the healthy control group (*P* = 0.00). Nevertheless, there were no significant differences in heart rate (*P* = 0.27), cardiac output (*P* = 0.55), and cardiac index (*P* = 0.70) between the groups. In contrast to other haemodynamic measures of cardiac function, cardiac power output was significantly higher in the healthy controls (*P* = 0.02). Oxygen consumption was significantly higher in heart failure (*P* = 0.05), while arteriovenous oxygen difference showed no significant difference.

**Table 2:** Resting haemodynamic and metabolic variables

	Healthy controls (*n* = 20)	HF patients (*n* = 17)	Percentage difference	*P*‐value
Heart rate (beats/min)	70.2 ± 8.5	67.5 ± 7.0	3.8	0.27
Systolic blood pressure (mmHg)	140 ± 15.4	117.8 ± 18.4	16.0	<0.01
Diastolic blood pressure (mmHg)	85.9 ± 9.1	74.3 ± 7.6	13.5	<0.01
Mean arterial blood pressure (mmHg)	104 ± 9.4	88.7 ± 8.0	14.7	<0.01
Cardiac output (L/min)	5.90 ± 1.0	6.1 ± 1.0	−3.3	0.55
Cardiac power output (W)	1.40 ± 0.2	1.20 ± 0.2	14.3	0.02
Cardiac index (L/min/m^2^)	3.20 ± 0.5	3.10 ± 0.5	3.1	0.70
Arteriovenous oxygen difference (mL/100 mL)	4.60 ± 0.5	5.30 ± 1.5	−13.2	0.08
Oxygen consumption (mL/kg/min)	3.60 ± 0.5	3.80 ± 1.2	−5.3	0.49

HF, heart failure.

### Difference in cardiac function at peak exercise

*Table* 3 shows the difference in haemodynamic and metabolic measures at peak exercise. In contrast to the results obtained at rest, the difference in haemodynamic variables between heart failure and healthy controls is more pronounced at peak exercise. The average value of cardiac power output was 36% higher in the healthy control group (5.0 ± 2.0 vs. 3.2 ± 1.2 W, *P* = 0.00). Oxygen consumption at peak exercise was significantly higher in the healthy control group, indicating better exercise tolerance compared with patients with heart failure. No significant differences between the groups were found for arteriovenous oxygen difference.

**Table 3:** Peak exercise haemodynamic and metabolic variables

	Healthy controls (*n* = 20)	HF patients (*n* = 17)	Percentage difference	*P*‐value
Heart rate (beats/min)	148 ± 23.7	111 ± 20.9	25.0	<0.01
Systolic blood pressure (mmHg)	202 ± 16.2	155 ± 13.4	23.1	<0.01
Diastolic blood pressure (mmHg)	94.4 ± 14.0	80.2 ± 8.5	15.0	<0.01
Mean arterial blood pressure (mmHg)	130 ± 11.5	105 ± 13.4	19.3	<0.01
Cardiac output (L/min)	18.2 ± 6.3	13.5 ± 4.0	25.8	0.01
Cardiac power output (W)	5.0 ± 2.0	3.2 ± 1.2	36.0	0.00
Cardiac index (L/min/m^2^)	9.8 ± 2.9	6.9 ± 1.7	29.6	0.00
Arteriovenous oxygen difference (mL/100 mL)	10.5 ± 2.3	10.8 ± 2.2	−2.8	0.62
Oxygen consumption (mL/kg/min)	24.3 ± 9.5	16.8 ± 3.8	30.1	<0.01

HF, heart failure.

### Relationship between NT‐proBNP and measures of cardiac function and performance

**Figure 1:**
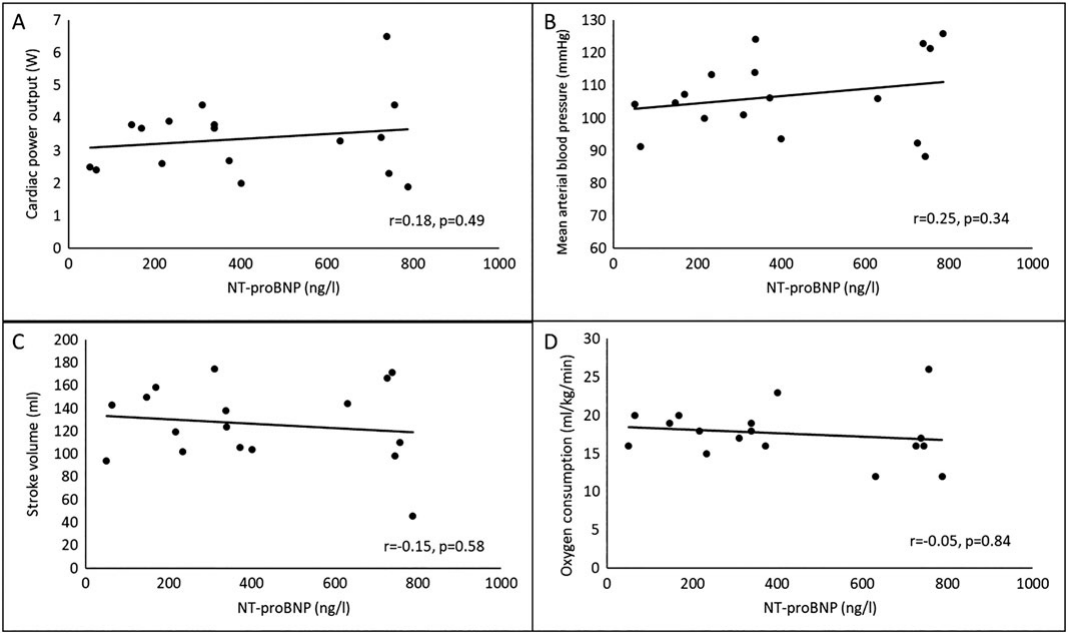
Relationship between N‐terminal prohormone of brain natriuretic peptide (NT‐proBNP) and peak exercise: cardiac power output (A), mean arterial blood pressure (B), stroke volume (C), and oxygen consumption (D).

*Table* 4 shows the relationship between NT‐proBNP and other variables of cardiac function and performance in patients with chronic heart failure. There is no significant relationship between NT‐proBNP and any haemodynamic and metabolic variables, neither at rest nor at peak exercise. *Figure* *1* shows the relationship between NT‐proBNP and peak exercise measures of cardiac performance.

**Table 4:** Correlation between N‐terminal prohormone of brain natriuretic peptide and measures of cardiac function and performance

	Rest (*n* = 17)			Peak exercise (*n* = 17)		
	*r*	*R* ^2^	*P*	*r*	*R* ^2^	*P*
Left ventricular ejection fraction (%)	0.07	0.01	0.79	—	—	—
Heart rate (beats/min)	0.45	0.21	0.07	0.45	0.21	0.07
Systolic blood pressure (mmHg)	−0.44	0.19	0.08	0.24	0.06	0.35
Diastolic blood pressure (mmHg)	0.09	0.01	0.73	0.14	0.02	0.58
Mean arterial blood pressure (mmHg)	−0.23	0.05	0.37	0.25	0.06	0.34
Stroke volume (mL)	−0.43	0.19	0.08	−0.15	0.02	0.58
Cardiac output (L/min)	−0.18	0.03	0.50	0.13	0.02	0.63
Cardiac power output (W)	−0.28	0.08	0.28	0.18	0.03	0.49
Cardiac index (L/min/m^2^)	−0.15	0.02	0.55	0.18	0.03	0.50
Arteriovenous oxygen difference (mL/100 mL)	0.03	0.00	0.92	−0.05	0.00	0.85
Oxygen consumption (mL/kg/min)	−0.18	0.03	0.50	−0.05	0.00	0.84

## Conclusion

The aim of the present study was to assess haemodynamic response to exercise and to evaluate the relationship between NT‐proBNP, cardiac function, and exercise tolerance in chronic heart failure.

The major findings of the present study suggest that (i) heart failure patients demonstrate a significantly diminished cardiac function (cardiac power output) at rest and in response to exercise and diminished exercise tolerance (peak oxygen consumption) compared with healthy controls and (ii) there is no significant relationship between the NT‐proBNP and measures of cardiac function and performance. It appears that there is a consensus among physicians based on their experience that natriuretic peptides should be considered with caution as an indicator of overall patients' symptoms and cardiac function.

The present study corroborates the findings of Bain and colleagues who demonstrated that patients with heart failure have a significantly lower peak exercise cardiac power output compared with healthy controls.^9^ However, the lack of significant relationship between NT‐proBNP and cardiac power output contrasts the findings of Williams and colleagues who suggested a significant, moderate negative relationship between the two measures (*r* = −0.64).^10^ The discrepancy between the results may be explained by the methodological limitations of the Williams *et al*. study, which may affect homogeneity of data and their distribution, i.e. they included patients with heart failure with preserved ejection fraction, classes I–IV of heart failure, as well as healthy individuals, which is demonstrated with significantly differing values of NT‐proBNP. Furthermore, the present study finding, which suggests lack of a significant relationship between NT‐proBNP and peak O_2_ consumption, differs from that of Felker and colleagues,^11^ who revealed a significant, moderate, negative relationship between the two variables (*r* = −0.39). Despite large sample size (*N* = 1383), lower age of patients (59 years old), and statistical significance (*P* < 0.0001), it should be acknowledged that only moderate strength of the relationship (*r* = 0.4) between does not warrant conclusion that NT‐proBNP is a strong/excellent predictor (or marker) of functional capacity, i.e. peak O_2_ consumption in chronic heart failure.

The two major limitation of the present study are (i) a small sample size and (ii) functional capacity of patients was a relatively preserved and NT‐proBNP rather elevated than high. These limitations may affect generalizability of the major findings.

In conclusion, results from the present study suggest the lack of significant and strong relationship between the NT‐proBNP and measures of cardiac function and exercise tolerance. It is reasonable to suggest that natriuretic peptides should be considered with caution in interpretation of the severity of cardiac dysfunction and functional capacity in chronic heart failure.

## Conflict of interest

None declared.

## Funding

This study was funded by the UK NIHR Newcastle Biomedical Research Centre grant to D.G.J. (grant no. BH161161). D.G.J. is supported by the UK Research Councils' Newcastle Centre for Ageing and Vitality (grant no. L016354). The views expressed are those of the authors and not necessarily those of the funding bodies. N.O. is supported by the European Horizon 2020 research and innovation programme (grant agreement no. 777204). The funders of the study had no role in study design or in data collection, analysis, or interpretation.
